# A Rare Case of Complete Heart Block and Takotsubo Cardiomyopathy in an Elderly Patient: A Case Report

**DOI:** 10.7759/cureus.62572

**Published:** 2024-06-17

**Authors:** Shaheen Rizly, Dhaval Trivedi

**Affiliations:** 1 Medicine, New York Presbyterian Brooklyn Methodist Hospital, Brooklyn, USA; 2 Internal Medicine, New York Presbyterian Brooklyn Methodist Hospital, Brooklyn, USA

**Keywords:** 3rd degree heart block, takotsubo cardiomyopathy (ttc), takotsubo and sinus node dysfunction, takotsubo cardioyopathy, cardiac arrhythmia and takotsubo cardiomyopathy

## Abstract

Although Takotsubo cardiomyopathy (TC) is often linked to various tachyarrhythmias, the coexistence of Takotsubo cardiomyopathy and complete heart block is rare, and the cause-and-effect relationship remains unclear. We present the case of an 83-year-old female with a history of known second-degree atrioventricular (AV) block who presented with syncopal episodes and bradycardia. She was diagnosed with a complete heart block requiring a dual-chamber pacemaker. Upon case review, transthoracic echocardiography revealed severe apical hypokinesis, prompting coronary angiography, which showed normal coronary arteries, consistent with Takotsubo cardiomyopathy. This case explores the relationship between Takotsubo cardiomyopathy and complete heart block, as well as the potential pathophysiological mechanisms involved.

## Introduction

Takotsubo cardiomyopathy (TC) is a subtype of non-ischemic cardiomyopathy characterized by transient left ventricular dysfunction [1]. Initially considered rare, recent epidemiological studies have revealed a higher prevalence than previously recognized, ranging from 2-3% of all suspected cases of acute coronary syndrome. Despite its transient nature, Takotsubo cardiomyopathy can lead to significant morbidity and mortality, with complications ranging from heart failure to life-threatening cardiogenic shock [2]. Upon echocardiography, Takotsubo cardiomyopathy typically presents with apical ballooning, although atypical variants of hypokinesis may be seen. The diagnosis of Takotsubo cardiomyopathy poses a challenge, as it is a diagnosis of exclusion and requires a coronary angiogram to rule out ischemic cardiomyopathy, as it often mimics their presentation [3]. However, coronary angiography cannot be performed in all patients with presumed Takotsubo syndrome, particularly in the oldest ones; therefore, a noninvasive approach comprehensive of the InterTAK Diagnostic Score assessment has recently been proposed for estimating the Takotubo syndrome probability in very old and frail patients with symptoms and signs suggestive of Takotsubo syndrome [4]. Treatment strategies primarily focus on supportive care, including the use of guideline-directed medical therapy for heart failure and diuresis for symptomatic relief. However, addressing the underlying stressors and providing psychological support are integral components in the management of Takotsubo cardiomyopathy [3].

The multifaceted nature of Takotsubo cardiomyopathy, involving sympathetic activation and myocardial stunning, contributes to the complexity and heterogeneity of arrhythmias observed in affected individuals. Takotsubo cardiomyopathy is associated with a spectrum of arrhythmias, including atrial fibrillation, atrial flutter, ventricular tachycardia, premature ventricular contractions (PVCs), and atrioventricular (AV) blocks [5]. The reported prevalence of atrial fibrillation in Takotsubo cardiomyopathy ranges from 5-15%, while ventricular arrhythmias were documented in 7.5% of affected patients [2,3,6]. An atrioventricular (AV) block refers to the disruption of electroconductive signals that are transmitted from the sinoatrial (SA) node to the ventricles through the atrioventricular (AV) node [7]. Third-degree AV block, also known as complete heart block, is a type of atrioventricular (AV) block that refers to the complete loss of transmission of electroconductive signals between the SA node and ventricles, which often occurs from a variety of etiologies, including structural heart disease, ischemic heart disease, medication-induced, and electrolyte abnormalities [7]. Conduction abnormalities, such as complete AV blocks, have been observed in Takotsubo cardiomyopathy. The prevalence of complete AV blocks in patients diagnosed with Takotsubo cardiomyopathy remains at only 2.8% [8]. While existing literature predominantly focuses on Takotsubo cardiomyopathy's association with various arrhythmias, little is known about the spectrum of conduction abnormalities in Takotsubo cardiomyopathy.

## Case presentation

**Figure 1:**
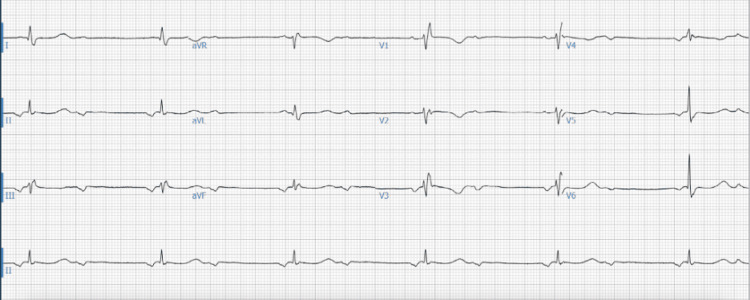
Bradycardia with a second-degree 2:1 A-V block and a right bundle branch block

An 83-year-old female with a past medical history of hypertension and second-degree AV block presented to the emergency department after being referred from her cardiologist's office for syncopal episodes and bradycardia. There, she was found to be in complete heart block with a right bundle branch block and left anterior fascicular escape. The patient endorsed having intermittent, sudden-onset syncopal episodes for one week without any precipitating factors or prodromal or postictal symptoms. Notably, the patient had a known intranodal conduction disease diagnosed six months ago. At that time, she was noted to have ectopic atrial or junctional bradycardia to 34 beats/minute (minimum heart rate 28 bpm) with frequent blocked premature atrial complexes (PACs), sinus blocks, and a 2:1 heart block (46%) (Figure 1). During that same time, a transthoracic echocardiogram (TTE) revealed a normal ejection fraction of 55-60% with no ventricular abnormalities. She was offered a permanent pacemaker (PPM) several times but declined.

**Figure 2:**
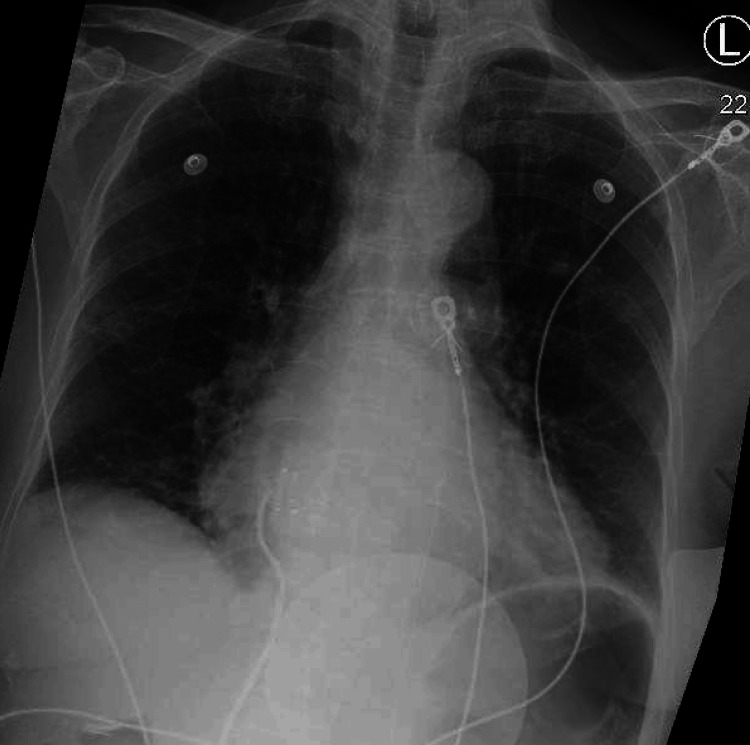
A chest roentgenogram on admission shows prominent upper zone vessels, an enlarged cardiac silhouette, and aortic arch calcifications

**Figure 3:**
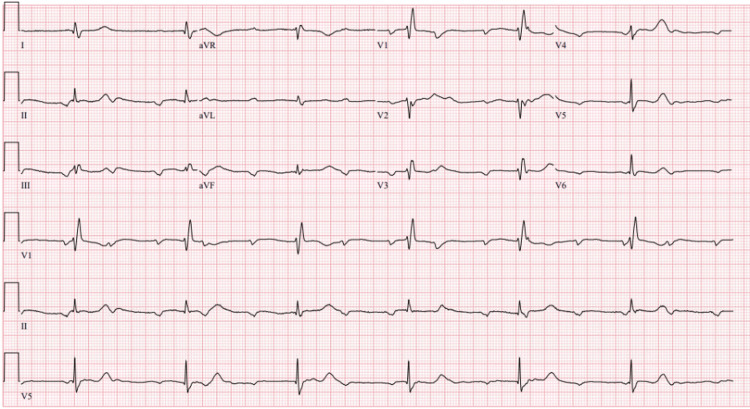
Bradycardia (heart rate 38) with complete heart block

In the emergency department, the patient's vital signs showed a heart rate of 39, blood pressure of 146/55, and arterial oxygen saturation of 96% on room air. On the physical exam, the patient was fully alert and oriented, with a cardiac exam revealing regular bradycardia and warm extremities. Labs revealed elevated troponins of 685 (normal < 14ng/L), elevated NT-BNP of 705 (normal < 450 pg/mL), and otherwise unremarkable metabolic and thyroid panels (Table 1). The chest X-ray demonstrated prominent upper zone vessels, an enlarged cardiac silhouette, and aortic arch calcifications (Figure 2). An EKG revealed a complete heart block (Figure 3).

**Table 1:** Initial lab values on admission (H): Data is abnormally high

Parameter tested	Reference Range & Units	Result
WHITE BLOOD CELL COUNT	3.40 - 11.20 x10^3/uL	8.74
HEMOGLOBIN	11.2 - 15.7 g/dL	13.5
HEMATOCRIT	34.1 - 44.9 %	38.8
MEAN CORPUSCULAR VOLUME (MCV)	80.0 - 100.0 fL	91.5
MEAN CORP HGB (MCH)	25.6 - 32.2 pg	31.8
MEAN CORP HGB CONC (MCHC)	32.2 - 36.5 g/dL	34.8
RED CELL DIST WIDTH (RDW)	11.6 - 14.4 %	13.5
RED BLOOD CELL COUNT	3.93 - 5.22	4.24
PLATELET COUNT, AUTO	150 - 450 x10^3/uL	176
MEAN PLATELET VOLUME, AUTO	9.4 - 12.4 fL	10.7
SODIUM	136 - 145 mmol/L	136
POTASSIUM	3.5 - 5.1 mmol/L	3.5
CHLORIDE	98 - 107 mmol/L	99
CARBON DIOXIDE	22 - 29 mmol/L	25
UREA NITROGEN (BUN)	8 - 23 mg/dL	8
BUN/CREATININE RATIO		17
CREATININE	0.51 - 0.95 mg/dL	0.51
GLUCOSE	74 - 106 mg/dL	109 (H)
ANION GAP	9 - 16 mmol/L	12
CALCIUM, TOTAL	8.2 - 10.2 mg/dL	9.1
MAGNESIUM (MCNC)	1.6 - 2.6 mg/dL	2.1
PROTEIN, TOTAL	6.6 - 8.7 g/dL	7
ALBUMIN, SERUM/PLASMA	3.5 - 5.2 g/dL	3.7
GLOBULIN	1.5 - 5.2 g/dL	3.3
BILIRUBIN, TOTAL	<=1.2 mg/dL	0.5
BILIRUBIN, DIRECT	<=0.3 mg/dL	<0.2
BILIRUBIN, INDIRECT	0.2 - 0.9 mg/dL	NCAL
ASPARTATE AMINOTRANSFERASE	10 - 35 U/L	51 (H)
ALANINE AMINOTRANSFERASE	10 - 35 U/L	30
ALKALINE PHOSPHATASE	35 - 104 U/L	98
B-TYPE NATRIURETIC (BNP), N-TERMINAL	<=450 pg/mL	705 (H)
TROPONIN-T, HIGH SENSITIVITY	<=14 ng/L	686 (H)
THYROTROPIN (TSH), 3RD GENERATION	0.3 - 4.2 uIU/mL	1.2

An echocardiogram at her primary cardiologist's office before admission revealed a normal left ventricular ejection fraction (55-60%) with mild pulmonary hypertension. The patient underwent successful implantation of a dual-chamber PPM in DDD mode. Post-procedural transthoracic echocardiography was performed and demonstrated mildly reduced left ventricular ejection fraction (45-50%) with severe left ventricular apical hypokinesis, severely dilated left atrium (left atrial volume index 61 mL/m²; normal range 16-35 mL/m²), moderate mitral regurgitation, and moderate aortic regurgitation (Video 1). The patient subsequently underwent coronary angiography, revealing no angiographic disease (Video 2, Video 3), elevated left ventricular end-diastolic pressure (LVEDP) of 20 mmHg, and no transvalvular gradient. 

**Video 1:** Transthoracic echocardiography (PLAX) demonstrating left ventricular apical hypokinesis PLAX: Parasternal long axis view

**Video 2:** Left heart catheterization: Right coronary artery

**Video 3:** Left heart catheterization: Left coronary artery

At this time, additional history was provided by the patient that revealed she was in a heightened state of emotional stress given the recent death of her husband. Given this supplemental information, the diagnosis of stress-induced Takotsubo cardiomyopathy was made, and the patient was gradually introduced to guideline-directed medical therapy (GDMT) for heart failure and eventually discharged home. Several months post-discharge, the PPM device was interrogated, which revealed a persistent underlying complete heart block rhythm.

## Discussion

The precise pathophysiological mechanisms linking Takotsubo cardiomyopathy to conduction disturbances, such as complete heart block, remain incompletely understood. However, several hypotheses have been proposed.

One potential mechanism involves the impact of excess catecholamine on the myocardium. Emotional stress can trigger a sympathetic surge, leading to the release of high levels of catecholamines. These catecholamines may result in the stunning of the myocardium, affecting both contraction and conduction. The catecholamine surge in Takotsubo cardiomyopathy may lead to direct myocardial injury, causing stunning and dysfunction. This could potentially extend to the conduction system, resulting in various arrhythmias, including a complete heart block [2,9]. 

Patients with pre-existing conduction abnormalities may be more vulnerable to the effects of sympathetic surges. Patients with Takotsubo cardiomyopathy and conduction abnormalities had a higher incidence of adverse events, including cardiogenic shock and mortality [10]. The synergistic impact of intrinsic conduction system disease and stress-induced catecholamine release could lead to a higher propensity for complete heart block in the setting of Takotsubo cardiomyopathy.

Neural and humoral factors beyond catecholamines may also contribute to the association between complete heart block and Takotsubo cardiomyopathy. The autonomic nervous system, including both sympathetic and parasympathetic branches, exerts a profound influence on the heart's electrical conduction. The intricate balance between these systems may be disrupted in the setting of acute stress, impacting both myocardial contractility and conduction. Studies exploring the neurohumoral mechanisms in Takotsubo cardiomyopathy have proposed that alterations in sympathetic and parasympathetic tone may create an environment conducive to arrhythmias, including heart block [11]. These findings suggest a multifaceted interplay between the autonomic nervous system and the conduction system, potentially contributing to the observed conduction abnormalities seen in Takotsubo cardiomyopathy.

The aforementioned mechanisms point towards Takotsubo cardiomyopathy as a possible etiology of conduction abnormalities. In this case, the patient had a known history of AV blockade with relatively unremarkable findings on her echocardiography six months before the development of Takotsubo cardiomyopathy and also had persistent complete heart block despite the initiation of guideline-directed medical therapy and permanent pacemaker implantation. This raises the possibility of her conduction abnormality acting as a trigger for the development of Takotsubo cardiomyopathy, which, however, could be challenged given the fact that Takotsubo cardiomyopathy commonly affects the mid or apical ventricular regions and spares the basal ventricular regions that contain the AV node.

Of note, it is also suspected that the recovery of myocardial contractility in Takotsubo cardiomyopathy may occur much quicker compared to the recovery of the cardiac conduction system, which could explain why several case reports demonstrate the persistence of conduction abnormalities long after the resolution of Takotsubo cardiomyopathy [12]. Therefore, the decision to implant a pacemaker in Takotsubo cardiomyopathy patients hinges on factors such as persistent conduction abnormalities, the severity of bradycardia-related symptoms, long-term monitoring, and the overall clinical status. Individualized approaches should be used, weighing the risks of untreated bradycardia-related complications against the potential reversibility of conduction abnormalities [2].

## Conclusions

The case highlights the rare but significant co-occurrence of complete heart block and Takotsubo cardiomyopathy. While Takotsubo cardiomyopathy is increasingly recognized, its association with complete heart block remains relatively uncommon. The pathophysiological mechanisms linking Takotsubo cardiomyopathy to complete heart block are not fully elucidated but likely involve catecholamine excess, neural-humoral factors, and the vulnerability of pre-existing conduction abnormalities. The sympathetic surge triggered by emotional stress may lead to direct myocardial injury, affecting both contraction and conduction, particularly in patients with underlying conduction system disease. Management of such cases necessitates a tailored approach, considering the severity of symptoms, the persistence of conduction abnormalities, and the overall clinical status. While pacemaker implantation may be warranted for symptomatic bradycardia, the decision should be weighed against the potential reversibility of conduction abnormalities and the patient's long-term prognosis. Moreover, addressing underlying stressors and providing psychological support are integral components of management, emphasizing the holistic care required for patients with Takotsubo cardiomyopathy. Further research is needed to explain the precise mechanisms underlying the association between Takotsubo cardiomyopathy and complete heart block, as well as to refine management strategies and improve outcomes for affected individuals. This case underscores the importance of comprehensive evaluation and individualized management in addressing the complex interplay between cardiac function and conduction abnormalities in patients with Takotsubo cardiomyopathy.
